# A single-cell sequencing-based prognostic model reveals HLA-DRA, NPC2, and PRPF38B as immune-regulatory tumor suppressors in non-small cell lung cancer

**DOI:** 10.3389/fcell.2026.1835398

**Published:** 2026-07-23

**Authors:** Wenya Su, Yudi Wu, Qian He, Lin Zhou, Jun Zhou

**Affiliations:** Department of Respiratory and Critical Care Medicine, The Third Affiliated Hospital of Soochow University, The First People’s Hospital of Changzhou, Changzhou, Jiangsu Province, China

**Keywords:** HLA-DRA, immune microenvironment, immune regulation, non-small cell lung cancer, NPC2, PRPF38B, risk score model, single-cell RNA sequencing

## Abstract

**Background:**

Non-small cell lung cancer (NSCLC) is the most common lung cancer subtype and a leading cause of cancer-related mortality due to asymptomatic early stages and poor 5-year survival, and tumor-infiltrating lymphocytes (TILs) play a pivotal but poorly defined role in NSCLC progression and prognosis within the tumor microenvironment (TME).

**Methods:**

We analyzed single-cell RNA sequencing data from GEO (GSE148466), NSCLC-related data from TCGA, and gene expression data from GEO (GSE50081). A 9-gene TIL-related risk score model (HLA-DRA, NPC2, PRPF38B, PABPC1, ENO1, ANXA2, LGALS1, S100A10, SRGN) was constructed using LASSO regression, with its prognostic value assessed via Kaplan-Meier and ROC analyses and external validation. Immune infiltration and regulatory factors were analyzed using TISIDB, pathway associations via GSVA and GSEA, and functional roles of key genes (HLA-DRA, NPC2, PRPF38 B) validated using RT-qPCR, Western blotting, and co-culture assays.

**Results:**

TILs were abundant in both smoking and non-smoking NSCLC samples. The 9-gene risk model effectively stratified patients by survival, with high-risk patients showing poorer outcomes and enriched tumor-promoting immune features and pathways. High-risk scores were associated with increased M0 macrophages, activated NK cells, and elevated immune checkpoints. Overexpression of HLA-DRA, NPC2, and PRPF38 B inhibited NSCLC cell proliferation, migration, and invasion, while enhancing apoptosis and suppressing M2 macrophage polarization.

**Conclusion:**

This study establishes a TIL-based prognostic risk model for NSCLC and identifies HLA-DRA, NPC2, and PRPF38 B as potential immune-regulatory therapeutic targets with tumor-suppressive functions, providing new insights for NSCLC precision immunotherapy.

## 1 Introduction

Non-small cell lung cancer (NSCLC) accounts for over 85% of all lung cancer cases and is a major cause of cancer-related mortality worldwide (Siegel et al., 2022). NSCLC exhibits slower growth and lower metastatic potential, yet its insidious early symptoms result in most patients being diagnosed at an advanced stage (Herbst et al., 2018; Travis et al., 2011). And even with the advancement of immunotherapy and targeted therapy, the 5-year survival rate remains unsatisfactory (Molina et al., 2008).

The occurrence and progression of NSCLC are closely associated with complex molecular mechanisms. Tumor cells, immune cells, fibroblasts, endothelial cells, and extracellular matrix make up the tumor microenvironment (TME), which is essential for controlling the growth of tumors (Quail et al., 2013). Among these components, immune cells, especially tumor-infiltrating lymphocytes (TILs), are essential in the immune regulation of NSCLC (Fridman et al., 2012).

Research has demonstrated that TILs contribute to the development and incidence of NSCLC in both directions. On one hand, TILs can activate antitumor immune responses to kill tumor cells, thereby inhibiting tumor growth and spread (Chen and Mellman, 2017). On the other hand, tumor cells can evade immune surveillance and weaken the killing ability of TILs, promoting immune tolerance to the tumor (Schreiber et al., 2011). Furthermore, the level and activity of TILs are closely related to the clinical prognosis of NSCLC patients. Patients with greater TIL levels have been shown to typically have higher survival rates (Remark et al., 2013).

Deeper knowledge of the variety and roles of TILs in NSCLC has been made possible by the advancement of single-cell sequencing technologies (Zheng et al., 2017). The activation status and makeup of TIL subpopulations fluctuate greatly amongst patients, according to single-cell RNA sequencing studies (Guo et al., 2018). For example, the proportion and functional status of CD8^+^ T cells and regulatory T cells (Tregs) in the tumor microenvironment significantly affect patients’ sensitivity to immunotherapy (Thommen and Schumacher, 2018). Additionally, the immune microenvironment and the results of NSCLC therapy are strongly correlated with the unique gene expression of TILs (Sade-Feldman et al., 2017). While many studies have explored the biological functions of TILs, their precise role in NSCLC remains to be further elucidated (Binnewies et al., 2018). Specifically, how to construct effective prognostic models using characteristic genes of TILs to guide personalized treatment is one of the current research hotspots (Wu et al., 2020).

To address these gaps, this study utilized single-cell RNA sequencing technology to explore the molecular mechanisms of TILs in NSCLC, identify TIL-associated key genes, and construct a reliable risk score prognostic model. We focused on the functional validation of HLA-DRA, NPC2, and PRPF38B, integrating bioinformatics analyses of multiple public databases (GSE148466, TCGA, GSE50081) and experimental validation to clarify their roles in regulating the immune microenvironment and tumor progression. This study aims to deepen the understanding of the NSCLC immune microenvironment at the molecular level, provide a novel prognostic model and potential therapeutic targets for NSCLC, and advance the development of precision immunotherapy strategies to improve patient outcomes and quality of life.

## 2 Materials and methods

### 2.1 Data download

We downloaded single-cell RNA sequencing (scRNA-seq) data from the GSE148466 dataset in the NCBI GEO public database (https://www.ncbi.nlm.nih.gov/geo/), which includes three samples with complete single-cell expression profiles for subsequent single-cell analysis. Meanwhile, we obtained the Series Matrix Files of the GSE50081 dataset from the NCBI GEO database. This dataset contains microarray-based gene expression data from 127 non-small cell lung cancer (NSCLC) patients, generated using the Affymetrix Human Genome U133 Plus 2.0 Array (platform GPL570). The GPL570 file provides probe annotation information (including probe sequences, gene IDs, and gene symbols) and was used to map microarray probe signals to corresponding genes in the GSE50081 dataset (Edgar et al., 2002).

Additionally, we retrieved bulk RNA sequencing data of 1,153 NSCLC patients from The Cancer Genome Atlas (TCGA, https://portal.gdc.cancer.gov/) (Chang et al., 2013), which provides multi-omics information such as mRNA, miRNA, and lncRNA expression data, DNA methylation, copy number variation, and SNPs. Both raw count data and normalized expression matrices were downloaded for downstream analyses.

### 2.2 Single-cell analysis

Samples with aberrant expression were filtered based on the proportion of mitochondrial genes, gene count, and UMI counts after the expression profiles were initially read using the Seurat program. The data were first normalized and standardized, and then subjected to linear dimensionality reduction using Principal Component Analysis (PCA). The optimal principal component (PC) number was observed using the elbow plot, followed by non-linear dimensionality reduction via UMAP to visualize the relationship between clusters. Cell types were annotated by referencing the CellMarker (Zhang et al., 2019) and panglaodb (Franzén et al., 2019) databases and literature to identify the corresponding cell types and their marker genes.

To identify genes specifically associated with TILs, we performed differential expression analysis between the TIL cluster (including CD8^+^ T cells, CD4^+^ T cells, and other lymphocyte subsets) and all other non-TIL cell types (epithelial cells, macrophages, fibroblasts, endothelial cells, plasma cells, mast cells, and ciliated cells) using the Wilcoxon rank-sum test as implemented in the Seurat FindMarkers function. Genes with |log_2_ (fold change)| ≥ 0.5 and adjusted p value (Benjamini–Hochberg false discovery rate, FDR) < 0.05 were considered significantly upregulated or downregulated in TILs. A total of 1,247 genes meeting these criteria were defined as TIL-associated genes and were subjected to further LASSO regression analysis for prognostic model construction.

### 2.3 Model construction and prognosis

Genes associated with TILs were first selected for further analysis. A prognostic model was then constructed using least absolute shrinkage and selection operator (LASSO) Cox regression with 10-fold cross-validation (nfold = 10). To avoid bias from a single random split, the TCGA dataset (1,153 NSCLC patients) was randomly split into training and testing sets at a ratio of 4:1 using random seeds ranging from 1 to 30,000. For each split, LASSO regression was performed on the training set using the glmnet R package (Simon et al., 2011), and the optimal penalty parameter λ was selected as lambda. min (the λ value that minimizes the mean cross-validated partial likelihood deviance). The final nine-gene signature was selected based on stable performance across multiple seeds, evaluated by C-index, AUC, and log-rank p-value in the training, internal testing, and external GEO validation sets.

Each patient’s risk score was then calculated based on the weighted regression coefficients from the LASSO analysis and the corresponding gene expression levels: RiskScore = Σ (coefficientᵢ × expressionᵢ). Patients were stratified into high-risk and low-risk groups according to the median risk score. Survival differences between the two groups were evaluated using Kaplan-Meier analysis and the log-rank test. The prognostic value of the risk score was further assessed by stratified analysis. The predictive accuracy of the model was evaluated using receiver operating characteristic (ROC) curves. All analyses were performed using the normalized bulk RNA-seq data of 1,153 NSCLC patients from the TCGA database.

### 2.4 Immune cell infiltration analysis

The CIBERSORT method is a widely used approach for evaluating immune cell types in the microenvironment. Based on the principle of support vector regression, this method performs deconvolution analysis on the expression matrix of immune cell subtypes. It includes 547 biomarkers that distinguish 22 human immune cell phenotypes, such as T cells, B cells, plasma cells, and myeloid cell subsets. Specifically, we used the LM22 signature matrix as the reference file, which contains the characteristic gene expression profiles of 22 immune cell subtypes. In this study, the CIBERSORT algorithm was applied to analyze patient data to infer the relative proportions of 22 types of immune-infiltrating cells (Newma et al., 2015). The running parameters were set as follows: number of permutations = 100 to estimate the significance of immune cell abundance, and quantile normalization (QN = TRUE) was enabled to normalize the mixed expression matrix. All other parameters were kept as default. For comparisons of immune cell fractions and immune regulatory gene expression levels between high- and low-risk groups, p-values were adjusted for multiple hypothesis testing using the Benjamini–Hochberg (BH) false discovery rate (FDR) method. Additionally, correlation analysis was conducted between gene expression levels and immune cell contents.

### 2.5 Drug sensitivity analysis

Drug sensitivity for each tumor sample was predicted using the Genomics of Drug Sensitivity in Cancer (GDSC) database (GDSC1 and GDSC2; https://www.cancerrxgene.org/), which contains drug response and genomic data for 970 (GDSC1) and 969 (GDSC2) cancer cell lines tested against 403 and 297 anticancer compounds, respectively. Normalized gene expression profiles of TCGA NSCLC patients were used as input for the predictive model. The R package pRRophetic was employed to estimate the IC50 values for each chemotherapeutic agent (Geeleher et al., 2014). Prediction accuracy was assessed using 10-fold cross-validation on the GDSC training set. All parameters were set to the default values, including averaging expression values when multiple probes corresponded to the same gene and applying the ComBat method to correct for batch effects.

### 2.6 GSVA analysis

Gene Set Variation Analysis (GSVA) was performed using the GSVA R package to evaluate differences in pathway activity between the high- and low-risk groups of TCGA NSCLC patients. The analysis was based on the normalized bulk RNA-seq expression profiles. Gene sets were obtained from the Hallmark (H) collection of the Molecular Signatures Database (MSigDB, version 7.5.1; https://www.gsea-msigdb.org/), which contains 50 curated gene sets representing well-defined biological states and processes (Liberzon et al., 2015).

### 2.7 GSEA analysis

Gene Set Enrichment Analysis (GSEA) was conducted using the clusterProfiler R package to identify enriched signaling pathways between the high- and low-risk groups. The Hallmark (H) and KEGG (C2) gene sets from the MSigDB database (version 7.5.1) were used as background reference sets. Enrichment results were considered significant when |NES| > 1, nominal p-value <0.05, and FDR <0.25.

### 2.8 miRNA network construction

After obtaining miRNAs associated with model genes from the miRcode database, the miRNA-gene network was displayed using Cytoscape.

### 2.9 Regulatory network analysis of key genes

The study used the “RcisTarget” R program to predict transcription factors. The overall number of motifs in the database determines the Normalized Enrichment Score (NES) of motifs, which serve as the foundation for all of RcisTarget’s calculations. In addition to the motifs annotated by the source data, this study also inferred further annotation files based on motif similarity and gene sequences. Finding each motif-gene pair’s area under the curve (AUC) was the first step in identifying overexpression of each motif in gene sets. The NES for each motif in the gene set was determined using the AUC distribution of each motif.

### 2.10 Nomogram model construction

A nomogram is a visual depiction of a regression analysis model that makes use of clinical symptoms and gene expression levels. On the same plane, scaled line segments depict the relationships between the variables in the prediction model. The magnitude of the regression coefficients, which indicate the contribution of each influencing component, was used to give scores to each value level of the influencing components in a multi-factor regression model. The predictive value was computed using the sum of the individual values to determine the overall score.

### 2.11 RT-qPCR

Following the manufacturer’s instructions, Trizol reagent (Beyotime, Beijing, China) was used to extract RNA from tissues. The extracted RNA (1 μg) was reverse-transcribed into cDNA using the Reverse Transcription Kit (Beyotime, Beijing, China, D7170S), and qPCR was performed using the SYBR Green qPCR Mix (Beyotime, Beijing, China, D7260) on an ABI 7900 fluorescence quantitative PCR apparatus (ABI, USA). The reaction began with a 10-min initial denaturation at 95 °C. It then went through 40 cycles, which included 10 s of denaturation at 95 °C, 15 s of annealing at 52.8 °C or 56.2 °C, and 20 s of extension at 72 °C. Prior to the first run, an automated gain calibration was used to apply a temperature ramp from 72 °C to 95 °C, rising by 1 °C per step. The 2^−ΔΔCT^ technique was used to calculate relative expression after normalizing mRNA expression to GAPDH. The list of primer sequences is as follows Table 1. All RT-qPCR experiments were performed with three independent biological replicates (cells from separate passages), and each biological replicate was measured in three technical replicates. Data are presented as mean ± SEM from the three biological replicates.

**TABLE 1:** Primer sequences.

Gene name	Species	Primers
HLA-DRA	Human	Forward primer: 5′-AGT​CCC​TGT​GCT​AGG​ATT​TTT​CA-3′
		Reverse primer: 5′-ACA​TAA​ACT​CGC​CTG​ATT​GGT​C-3′
NPC2	Human	Forward primer: 5′-CAA​AGG​ACA​GTC​TTA​CAG​CGT-3′
		Reverse primer: 5′-GGA​TAG​GGC​AGT​TAA​TTC​CAC​TC-3′
PRPF38 B	Human	Forward primer: 5′-TTAAGGTCACGCACGTTGAAC-3′
		Reverse primer: 5′-CCC​ATC​ACT​TGC​TTT​CGA​GTT​A-3′
Arginase-1	Human	Forward primer: 5′-TGG​ACA​GAC​TAG​GAA​TTG​GCA-3′
		Reverse primer: 5′-CCA​GTC​CGT​CAA​CAT​CAA​AAC​T -3′
GAPDH	Human	Forward primer: 5′-GGA​GCG​AGA​TCC​CTC​CAA​AAT-3′
		Reverse primer: 5′-GGC​TGT​TGT​CAT​ACT​TCT​CAT​GG-3′

### 2.12 Western blot

First, use RIPA buffer (BestBio, BR0002) to lyse the cells after removing the supernatant from the treated cell culture medium. The BCA Protein Assay Kit was used to quantify the amount of protein (Beyotime, P00012, Beijing, China). After blocking the proteins on the PVDF membrane with 5% non-fat milk, the primary antibody was incubated at 4 °C for the entire night. Primary antibodies include HLA-DRA (proteintech,17221-1-AP, 1:2000),NPC2(19888-1-AP, 1:1,000),Arginase-1 Polyclonal antibody (16001-1-AP, 1:5,000),PRPF38 B Polyclonal antibody (23901-1-AP, 1:1,000),GAPDH (10494-1-AP, 1:10,000). After washing the membrane the next day, the corresponding secondary antibody (A0201, 1:2000, Beyotime, Beijing, China) was added and incubated on a shaking platform at room temperature for 1.5 h. The blots were analyzed using enhanced chemiluminescence (ECL). Protein levels were quantified using ImageJ (V 15.0, NIH, USA). All Western blot experiments were performed with three independent biological replicates (cells from separate passages), and each biological replicate was run in three technical replicates (separate gels).

### 2.13 Immunofluorescence detection

First, deparaffinization and rehydration were performed by sequential treatment with xylene and ethanol solutions of varying concentrations. Subsequently, antigen retrieval was carried out by placing the tissue sections in antigen retrieval solution, followed by microwave treatment. The slides were allowed to cool and then washed with PBS. Next, the slides were blocked with blocking solution and incubated for 30 min at room temperature. Thereafter, the sections were incubated with primary antibodies (HLA-DRA at 1:200, NPC2 at 1:200, PRPF38 B at 1:200, and ARG1 at 1:200) overnight at 4 °C, after which the slides were washed. The sections were then incubated with the secondary antibody at room temperature for 1.5 h, followed by washing of the slides. Finally, mounting was performed by applying DAPI Fluoromount-G and covering with a coverslip. The slides were stored in the dark, and images were captured for analysis.

### 2.14 CCK8 assay for cell viability

The CCK-8 kit (Beyotime, C0038, Beijing, China) was used to conduct the CCK-8 test. After being sown in plates, cardiac cells were left to adhere. For the cell proliferation assay, 100 μL of 2000 cells was added per well. Following treatment under various experimental circumstances, each well received 10 μL of CCK-8 solution. The absorbance at 450 nm was determined after the cells were cultured for 1.5 h.

### 2.15 EdU assay

Cell proliferation was assessed using an EdU assay kit (C0071L, Beyotime, Beijing, China). After plating and treatment, cells were incubated with EdU working solution for 2 hours, then permeabilized, fixed, treated with click buffer, and stained with DAPI. To calculate the cell growth rate, fluorescence pictures were taken and examined using ImageJ software (Version 1.8.0, NIH, Bethesda, Maryland, USA).

### 2.16 Transwell assay

In the Transwell assay, H1299 and HCC827 cells were transfected with pc-NC, pc-HLA-DRA, pc-NPC2, and pc-PRPF38 B plasmids. Serum-free media (Beyotime, C0891) was used to seed cells in the top chamber of a Transwell insert (Corning). 10% fetal bovine serum was present in the medium in the lowest chamber (BasalMedia, S660JJ). Cells were cultured for twenty-four hours at 37 °C with 5% CO_2_. A cotton swab was used to remove any non-migrated cells following incubation. 0.1% crystal violet was used for 30 min to stain the migrating cells on the membrane following fixation with 4% paraformaldehyde. For the invasion assay, Matrigel (Corning) was applied to the upper chamber before performing the same procedure. Microscope images were taken, and migrated cells counted. Statistical analysis was then performed to compare the migration and invasion abilities among the different groups.

### 2.17 Flow cytometric analysis of cell apoptosis

To analyze cell apoptosis, a cell suspension was prepared and centrifuged at 500 *g* and 4 °C for 5 min to collect the cells. The cells were washed twice with pre-cooled PBS, with centrifugation performed under the same conditions each time. They were then gently resuspended in pre-cooled 1×Binding Buffer to adjust the concentration to 1–5 × 10^6^/mL. To 100 μL of the suspension, 5 μL Annexin V-FITC(G1511-50T, Servicebio, Wuhan, China) and 5 μL PI(G1021,Servicebio, Wuhan, China) were added, followed by gentle mixing. The mixture was incubated at room temperature in the dark for 8–10 min. Finally, 400 μL of pre-cooled 1×Binding Buffer was added, the mixture was gently mixed again, and analysis was performed using flow cytometry (Beckman, Cytoflex) within one hour.

### 2.18 Measurement of TGF-β, IL-10 and IL-13 by ELISA

Cell culture supernatants were collected at 48 h after cell transfection, and centrifuged at 12,000 *g* and 4 °C for 10 min to remove cell debris and insoluble impurities; the supernatant was collected and stored at −80 °C for subsequent detection. The levels of TGF-β (PT878, Beyotime, Beijing, China), IL-10 (PI522, Beyotime, Beijing, China), and IL-13 (PI539, Beyotime, Beijing, China) in cells were measured. An absorbance assessment was conducted utilizing a microplate reader (Kayto, RT6000).

### 2.19 Statistical analysis

Data analysis was performed through SPSS V 20 (IBM Corporation, Chicago, IL, USA). When comparing two groups, a t-test was utilized. The LSD test was used after a one-way ANOVA for comparisons involving three or more groups. All experiments were performed with three independent biological replicates, and each biological replicate contained three technical replicates. Data are presented as mean ± SEM from the three biological replicates. Statistical analyses were conducted using the values from the biological replicates (averaging technical replicates per biological replicate). For multiple comparisons in the immune infiltration analysis, p-values were adjusted using the Benjamini–Hochberg procedure to control the false discovery rate (FDR) at 0.05. All tests were two-tailed, with a p-value threshold of less than 0.05 was set to determine statistical significance.

## 3 Results

### 3.1 Revealing the cellular composition of the tumor microenvironment in NSCLC through single-cell analysis

**FIGURE 1:**
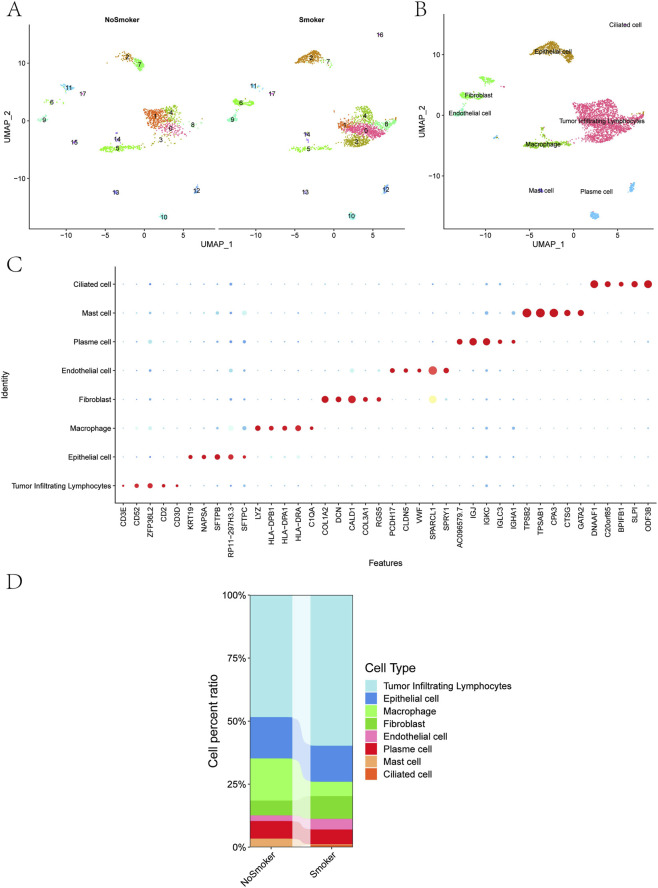
Single-Cell Analysis of Immune Microenvironment in NSCLC. **(A)** UMAP plot displaying cell type distribution in non-smokers and smokers. **(B)** Refined UMAP plot showing major cell type clusters. **(C)** Bubble plot of characteristic gene expression across cell types, with bubble size indicating expression levels and color representing significance. **(D)** Stacked bar chart showing the proportional distribution of cell types in non-smokers vs. smokers.

We integrated multiple data sources and used bioinformatics tools for standardized analysis and cell annotation. Single-cell data from GSE148466 (three patients), bulk RNA-seq data from GSE50081 (127 NSCLC patients), and TCGA (1,153 patients) were downloaded. Seurat was used to process the scRNA-seq data, retaining 4,080 high-quality cells after quality control. Violin and scatter plots visualized the data (Supplementary Figures S1A, S1B), and the top 10 highly variable genes were shown (Supplementary Figure S1C). Data normalization and integration were performed, followed by batch correction using Harmony; additionally, PCA was applied for dimensionality reduction to visualize the data (Supplementary Figures S1D-S1F). UMAP clustering identified 18 cell clusters (Figure 1A), which were annotated into eight cell types: Tumor Infiltrating Lymphocytes, Epithelial cells, Macrophages, Fibroblasts, Endothelial cells, Plasma cells, Mast cells, and Ciliated cells (Figure 1B). Bubble plots displayed marker gene expression (Figure 1C). Group analysis showed Tumor Infiltrating Lymphocytes dominated in both smokers (∼60%) and non-smokers (∼50%), with a higher prevalence in smokers (Figure 1D), highlighting their key role in the NSCLC immune microenvironment.

### 3.2 Construction of a risk score model based on LASSO regression and its application in prognostic prediction of NSCLC

**FIGURE 2:**
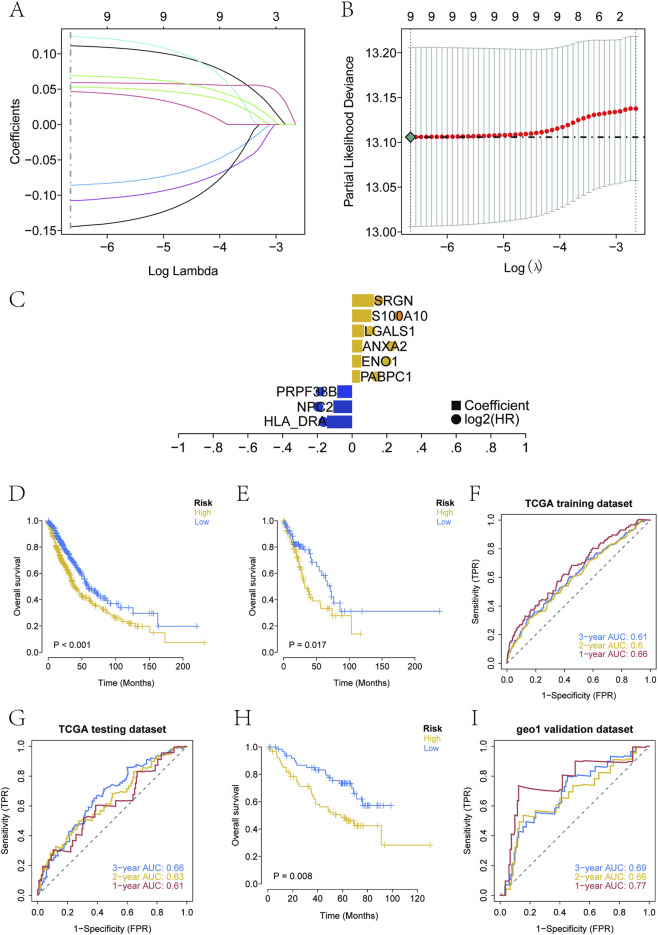
Risk Score Model Construction and Prognosis Prediction for NSCLC. **(A)** Coefficient path plot of LASSO regression showing gene coefficients at various Lambda values. **(B)** Cross-validation curve of LASSO, with optimal Lambda indicated by the red dot. **(C)** Bar chart of key genes selected by LASSO, with regression coefficients and log2(HR) values, colored by correlation. **(D,E,H)** Kaplan-Meier survival curves comparing high-risk and low-risk groups in TCGA **(D)**, test set **(E)**, and GEO validation set **(H)**. **(F,G,I)** ROC curves assessing model performance for 1-, 3-, and 5-year survival in TCGA **(F)**, test set **(G)**, and GEO validation set **(I)**.

We used LASSO regression to identify key NSCLC-related genes based on TIL-associated genes from the previous step. TCGA data with survival information were randomly split (4:1) into training and testing sets. Nine feature genes were selected via LASSO in the training set (Figures 2A,B), and a risk score model was built: RiskScore = HLA-DRA × (−0.1446) + NPC2 × (−0.1078) + PRPF38B × (−0.0859) + PABPC1 × 0.0467 + ENO1 × 0.0531 + ANXA2 × 0.0592 + LGALS1 × 0.0691 + S100A10 × 0.1115 + SRGN × 0.1246 (Figure 2C). Kaplan-Meier analysis showed significantly worse OS in the high-risk group (Figures 2D,E, P < 0.05). ROC analysis yielded AUC values ranging from 0.65 to 0.71 for 1-, 3-, and 5-year survival, indicating moderate but statistically significant discriminative ability (Figures 2F,G). To validate stability, we applied the model to GSE50081. The high-risk group again showed lower OS (Figure 2H, P < 0.05), with strong predictive power confirmed by ROC (Figure 2I). In summary, LASSO identified 9 genes to construct a robust risk model distinguishing high- and low-risk NSCLC patients with reliable prognostic value.

### 3.3 Analysis of the correlation between risk scores and clinical indicators in NSCLC prognosis

We analyzed risk score distribution across clinical markers including Distant Metastasis (M), Fustat, Gender, Stage, Lymph Node Metastasis (N), Tumor Size (T), and Age. The risk score was significantly higher in deceased patients (p = 1.575e-08; Supplementary Figure S2A), increased with tumor invasiveness (p = 3.752e-10; Supplementary Figure S2B), and was slightly higher in males (p = 2.515e-04; Supplementary Figure S2C). Higher clinical stage and N stage were also associated with higher risk scores (p = 7.628e-04 and p = 3.697e-03; Supplementary Figures S1D-S1E). However, no significant association was found with M stage or age (p = 0.697, 0.796; Supplementary Figures S1F-S1G).

Univariate analysis showed the risk score was a significant prognostic factor (p < 0.001, HR > 1; Supplementary Figure S2H), and multivariate analysis confirmed it as an independent predictor (p < 0.001; Supplementary Figure S2I), underscoring its clinical relevance in NSCLC prognosis.

### 3.4 Potential mechanism by which the risk score regulates the immune microenvironment to influence NSCLC progression

**FIGURE 3:**
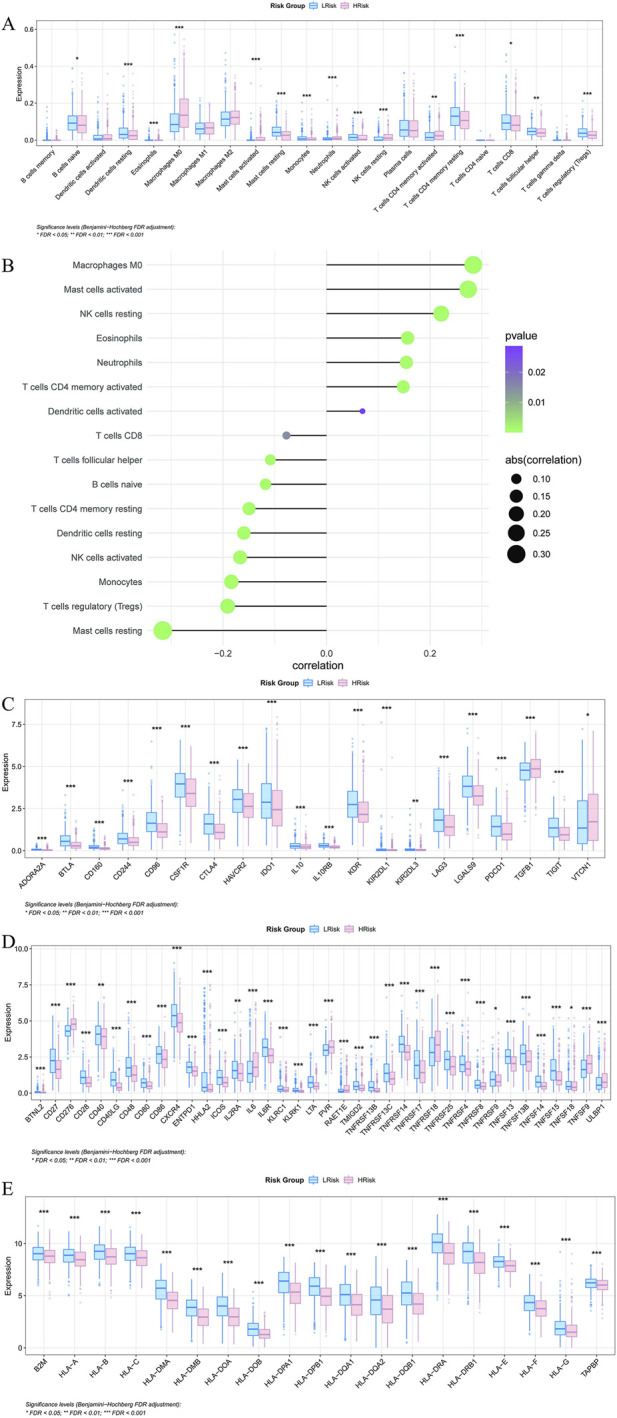
Immune characteristics associated with the risk model. **(A)** Boxplot displaying the distribution of different immune cell types in the high-risk and low-risk group samples. **(B)** Dot plot illustrating the correlation between immune cell types and risk score, where the size of the dots represents the correlation coefficient, and the color indicates the significance level (p-value). **(C,E)** Boxplots showing the expression levels of immune regulation-related genes in the high-risk and low-risk groups. ***p < 0.001, **p < 0.01, *p < 0.05 vs. LRisk.

The tumor microenvironment comprises cancer cells, immune cells, extracellular matrix, growth factors, inflammatory mediators, and tumor-associated fibroblasts. To explore NSCLC progression mechanisms, we analyzed the correlation between risk score and immune infiltration. Immune cell composition differed between risk groups (Supplementary Figure S3A), with activated NK cells, CD8 T cells, and resting CD4 memory T cells reduced in the high-risk group, while Macrophages M0 and activated CD4 memory T cells were elevated (Figure 3A). Risk score positively correlated with Macrophages M0 and activated CD4 memory T cells, but negatively with resting mast cells and activated NK cells (Figure 3B).

Using TISIDB data, we compared immune regulatory gene expression. High-risk patients showed elevated immune suppressors (CTLA4, PDCD1, TGFBR1) and stimulators (CD276, IL6), indicating stronger immune activation. PD-1 (encoded by PDCD1) and CTLA-4 are well-established immune checkpoint molecules that mediate T cell exhaustion in the tumor microenvironment (Wher et al., 2015). Chemokine CXCL8 and receptor CCR5 also varied significantly (Supplementary Figures S3B,3C; Figures 3C–E), suggesting altered immune recruitment. Overall, risk score is closely tied to immune infiltration and regulation, impacting NSCLC prognosis.

### 3.5 Risk score predicts chemotherapy drug sensitivity and its relationship with key molecular pathways in early NSCLC

**FIGURE 4:**
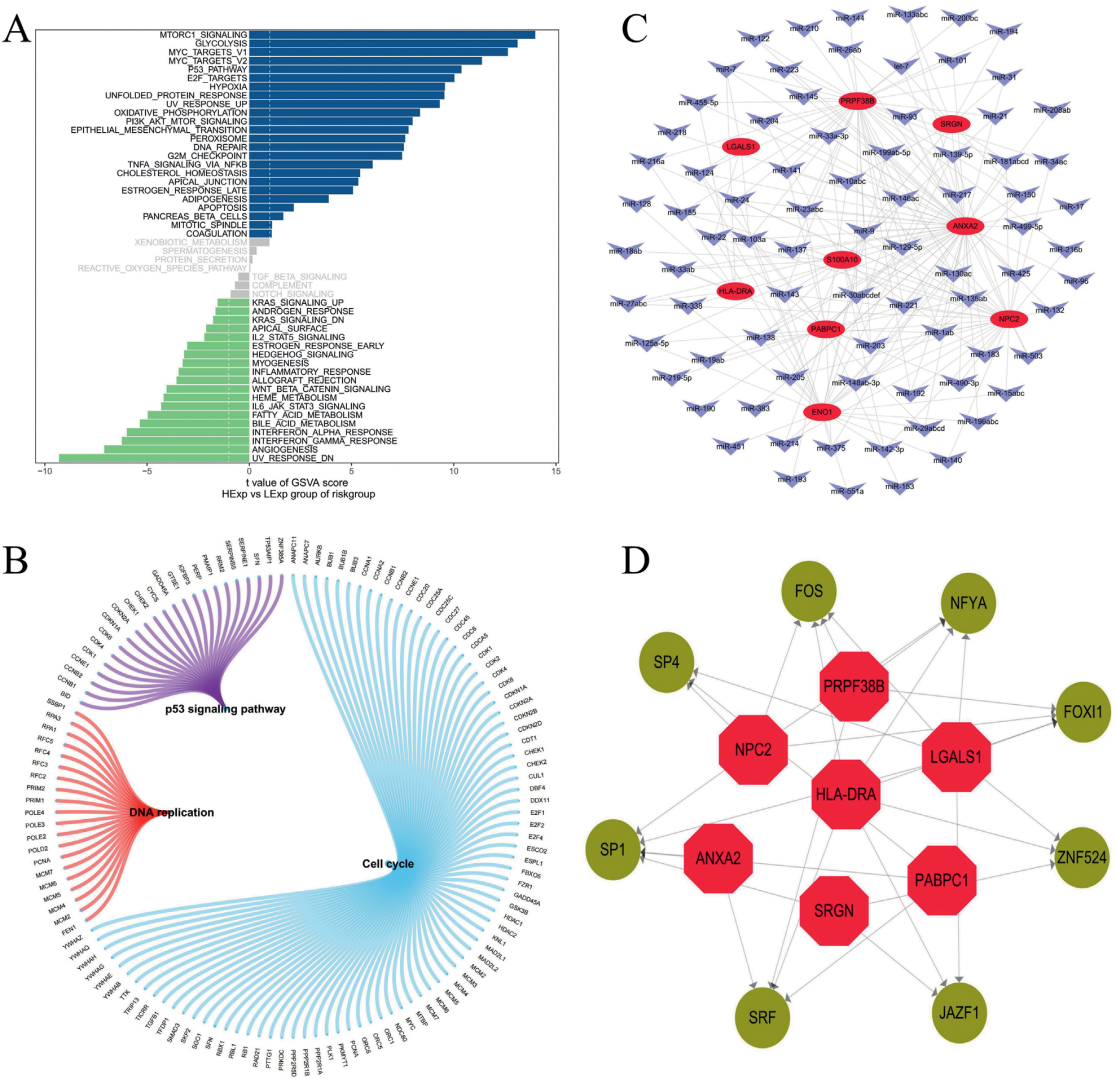
Functional Pathway Analysis and Regulatory Network Construction for NSCLC Risk Model. **(A)** Bar plot presenting the results of differential signaling pathway analysis between high-risk and low-risk groups based on GSVA (Gene Set Variation Analysis). **(B)** Molecular interaction network diagram illustrating the relationships between significantly enriched key pathways. The p53 signaling pathway (purple), DNA replication (red), and cell cycle (cyan) are highlighted. **(C)** A regulatory network constructed between the model genes and miRNAs. Red nodes represent core genes, and blue nodes represent miRNAs. Each line indicates a predicted regulatory relationship between a model gene and an miRNA. **(D)** The regulatory network between model genes (red nodes) and related transcription factors (green nodes).

Surgery combined with chemotherapy is effective for early-stage NSCLC. Using the R package “pRRophetic” and GDSC database data, we estimated chemotherapy sensitivity for each tumor sample and analyzed its correlation with the risk score. Sensitivity to axitinib, cytotarabine, AKT inhibitor VIII, BAY.61.3606, BIBW2992, and BMS.708,163 was significantly associated with the risk score (p < 0.05) (Supplementary Figure S4A). GSVA revealed differential enrichment of MTORC1-SIGNALING, P53-PATHWAY, and PI3K-AKT-MTOR-SIGNALING between high- and low-risk groups (Figure 4A), indicating their roles in tumor survival, proliferation, DNA repair, and apoptosis. The p53 signaling pathway is a central regulator of apoptosis, DNA repair, and cell cycle arrest, and its activation can suppress tumor progression (Vousde and Prives, 2009). The PI3K-AKT-mTOR signaling pathway controls cell growth, metabolism, and survival, and its aberrant activation is frequently observed in cancer (Glaviano et al., 2023).

GSEA further confirmed significant enrichment of the p53 pathway, DNA replication, and cell cycle regulation in the high-risk group (Supplementary Figure S4B), highlighting their roles in cell division and tumor growth. A molecular interaction network illustrating the connections between the nine model genes and these key signaling pathways is shown in Figure 4B. Overall, risk score correlates with chemotherapy sensitivity and key tumor-related signaling pathways, particularly p53, DNA replication, and cell cycle, underscoring its clinical relevance in early NSCLC.

### 3.6 Non-coding RNA network and transcription factor regulation of NSCLC model genes

We used the miRcode database to identify non-coding RNA networks linked to the 9 model genes (red nodes), predicting 80 miRNAs and 214 interactions, visualized via Cytoscape (Figure 4C). Motif analysis showed these genes are regulated by several transcription factors (TFs) (Figure 4D). The top enriched motif was cisbp-M3628, with TF NFYA showing the highest NES (6.79), indicating strong regulatory involvement (Supplementary Figure S4B).

### 3.7 Application and evaluation of the nomogram model based on risk scores for long-term survival prediction in NSCLC

**FIGURE 5:**
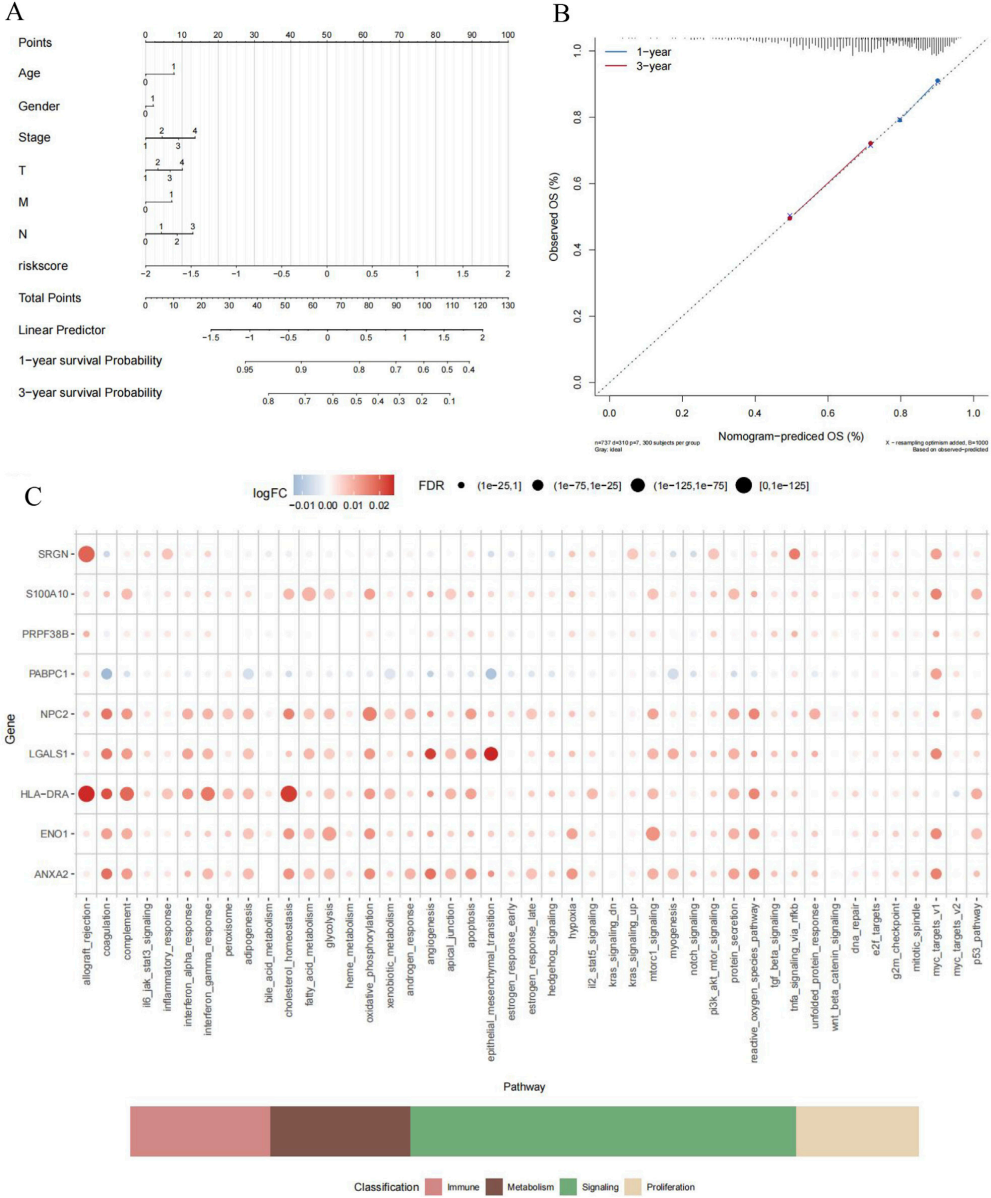
Nomogram Construction, Calibration, and Functional Annotation of Key Genes for NSCLC Risk Model. **(A)** The nomogram constructed based on risk scores, displaying the 1-year, 3-year, and 5-year survival probabilities for patients. The nomogram integrates clinical features such as age, gender, and stage. **(B)** Calibration curve evaluating the accuracy of the nomogram in predicting the 3-year survival probability. The x-axis represents predicted probabilities, and the y-axis represents actual survival probabilities. **(C)** Association analysis between model genes and Hallmark pathways. Single-cell AUCell was used to calculate pathway enrichment scores for each cell using the Hallmark gene set collection. For each of the nine model genes, cells were divided into high-expression (Hexp) and low-expression (Lexp) groups based on the median expression level of that gene. Differential pathway enrichment between Hexp and Lexp was then analyzed using the limma package. The vertical axis represents the nine model genes; the horizontal axis represents Hallmark pathways (grouped into immune, metabolic, signaling, and proliferation categories). Dot size is inversely proportional to the FDR after Bonferroni correction (larger dots indicate more significant differences, i.e., smaller FDR). Dot color represents the logFC (red: positive, blue: negative, white: no change). The results show that HLA-DRA is mainly negatively enriched in immune-related pathways, NPC2 is positively enriched in metabolic pathways, and PRPF38 B is positively enriched in proliferation-associated pathways.

We used regression analysis to build a nomogram after dividing samples into high- and low-risk groups based on the median risk score. The risk score had the highest weight, indicating its dominant predictive power (Figure 5A). Predictive analysis at 1- and 3-year time points showed strong agreement between predicted and actual outcomes (Figure 5B). ROC curves confirmed good discrimination, with high AUCs for 2- and 3-year survival (0.6053 and 0.6220) (Supplementary Figure S5A). Decision Curve Analysis (DCA) showed the risk score yielded high net benefit across most thresholds (Supplementary Figure S5B). Overall, the risk score is a strong predictor of long-term survival.

### 3.8 Expression characteristics of model genes in the NSCLC microenvironment and their potential association with tumor-related pathways

We analyzed the expression of nine model genes across 8 cell types (Supplementary Figure S5C-E). HLA-DRA was highly expressed in immune cells (particularly in TILs and macrophages), consistent with its canonical role in antigen presentation. NPC2 showed enrichment in cells with active lipid and cholesterol metabolism (e.g., macrophages and certain epithelial cell subsets), reflecting its function in cholesterol transport and intracellular cholesterol homeostasis. PRPF38B, a pre-mRNA splicing factor involved in spliceosome function, was detected across multiple cell types (including immune cells, epithelial cells, and fibroblasts), consistent with its role in fundamental RNA processing that is broadly required across diverse cell lineages. AUCell analysis showed HLA-DRA enriched in immune pathways (e.g., antigen presentation), NPC2 in metabolic pathways (e.g., lipid metabolism), and PRPF38 B in RNA processing (Figure 5C). These results highlight the distinct functions of model genes in the tumor microenvironment, metabolism, and immune regulation.

### 3.9 Downregulation of HLA-DRA, NPC2, and PRPF38 B in NSCLC cell lines

**FIGURE 6:**
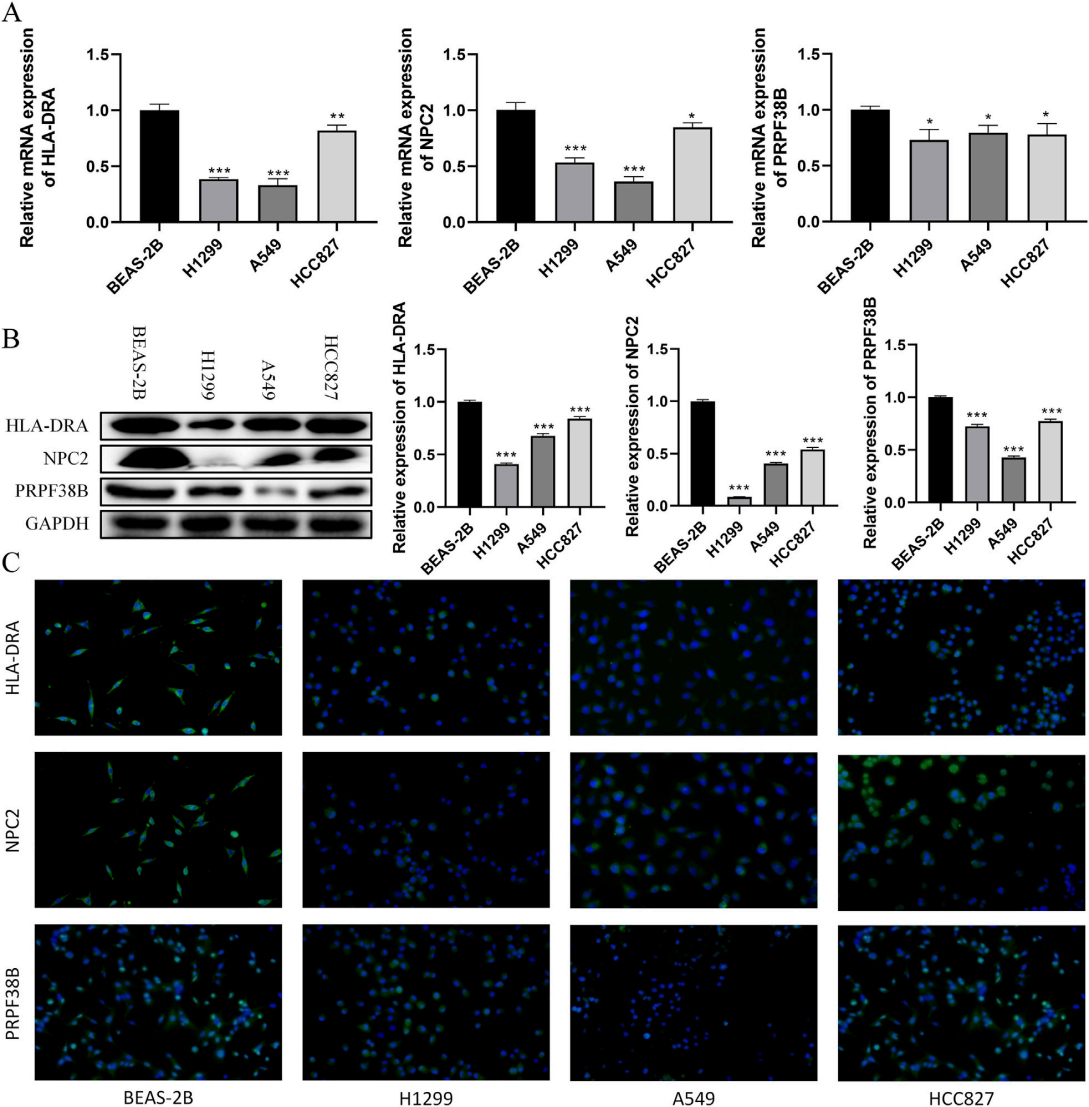
Downregulation of HLA-DRA, NPC2, and PRPF38B in Non-Small Cell Lung Cancer Cell Lines. **(A)** Bar chart showing the relative mRNA expression levels of HLA-DRA, NPC2, and PRPF38 B in BEAS-2B, H1299, A549, and HCC827 cell lines. **(B)** Western blot analysis detecting the protein expression levels of HLA-DRA, NPC2, and PRPF38 B in BEAS-2B, H1299, A549, and HCC827 cell lines. The bar chart on the right represents the relative protein expression levels of the corresponding proteins. **(C)** Immunofluorescence staining showing the localization and fluorescence intensity of HLA-DRA, NPC2, and PRPF38 B in BEAS-2B, H1299, A549, and HCC827 cells. ***p < 0.001, **p < 0.01, *p < 0.05 vs. HLA_DRA. The data are presented as mean ± SEM, and all experiments were performed in triplicate.

Based on previous research, HLA-DRA, NPC2, and PRPF38 B were selected as the primary focus for further investigation, as they may exert protective effects in NSCLC and have been less studied. First, the expression of HLA-DRA, NPC2, and PRPF38 B mRNA and protein levels were examined in normal lung epithelial cells (BEAS-2B) and NSCLC cell lines (H1299, A549, HCC827) using RT-qPCR and Western blotting (Figures 6A,B). In NSCLC cell lines, the results showed that HLA-DRA, NPC2, and PRPF38 B mRNA and protein levels were typically downregulated, especially in H1299 and A549 cells. Immunofluorescence results also confirmed this downregulation (Figure 6C).

### 3.10 Effects of overexpressing HLA-DRA, NPC2, and PRPF38 B on NSCLC cell proliferation, migration, invasion, and apoptosis

**FIGURE 7:**
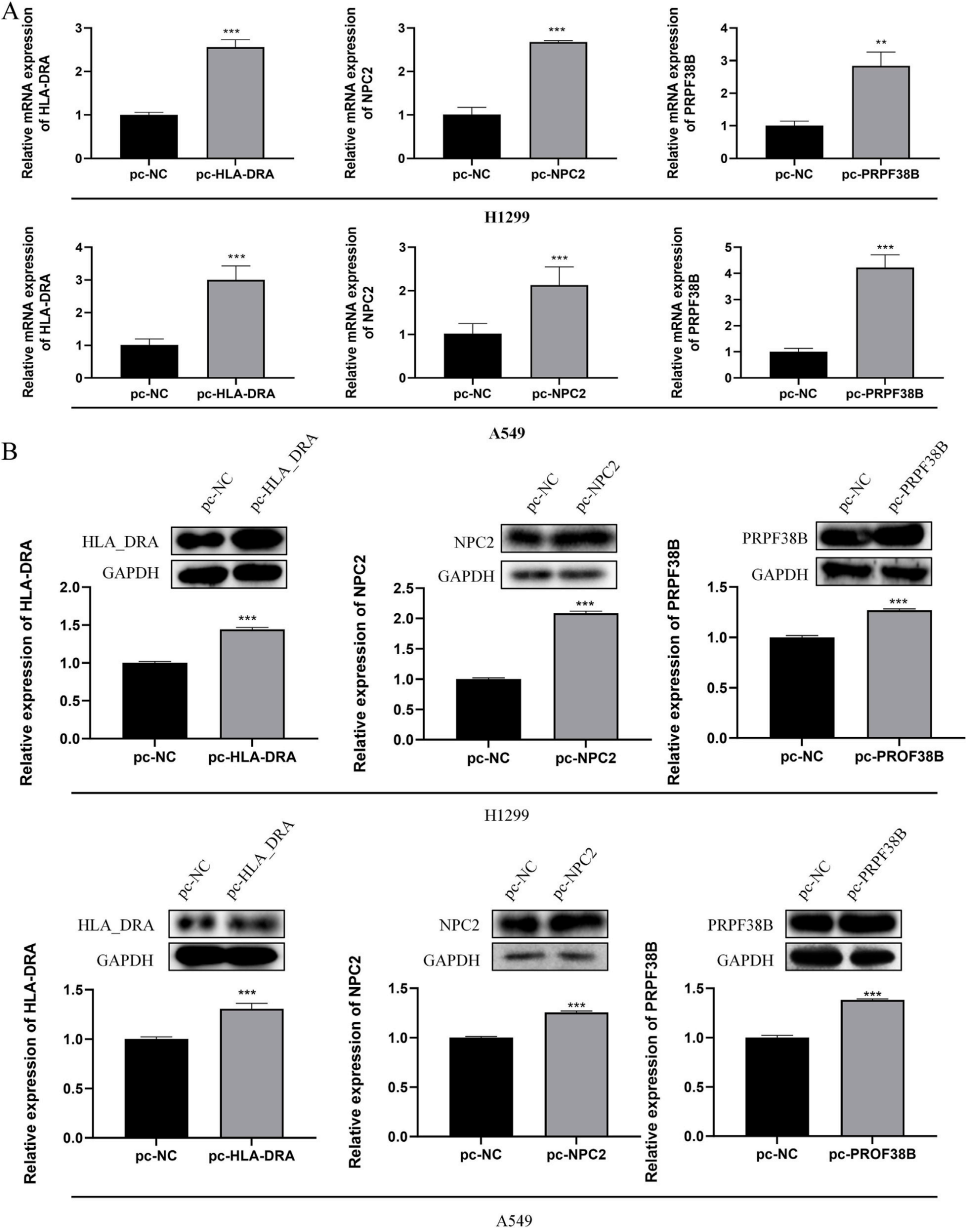
Overexpression Efficiency of HLA-DRA, NPC2, and PRPF38 B in H1299 and A549 Cells. **(A)** Bar chart showing the relative mRNA expression levels of HLA-DRA, NPC2, and PRPF38 B in H1299 and A549 cells transfected with pcDNA3.1 overexpression vectors (pc-HLA-DRA, pc-NPC2, and pc-PRPF38 B). **(B)** Western blot analysis detecting the protein expression levels of HLA-DRA, NPC2, and PRPF38 B in H1299 and A549 cells transfected with pcDNA3.1 overexpression vectors. The bar chart on the right represents the relative protein expression levels. ***p < 0.001, **p < 0.01 vs. pc-NC. Data are presented as mean ± SEM, and all experiments were performed in triplicate.

**FIGURE 8:**
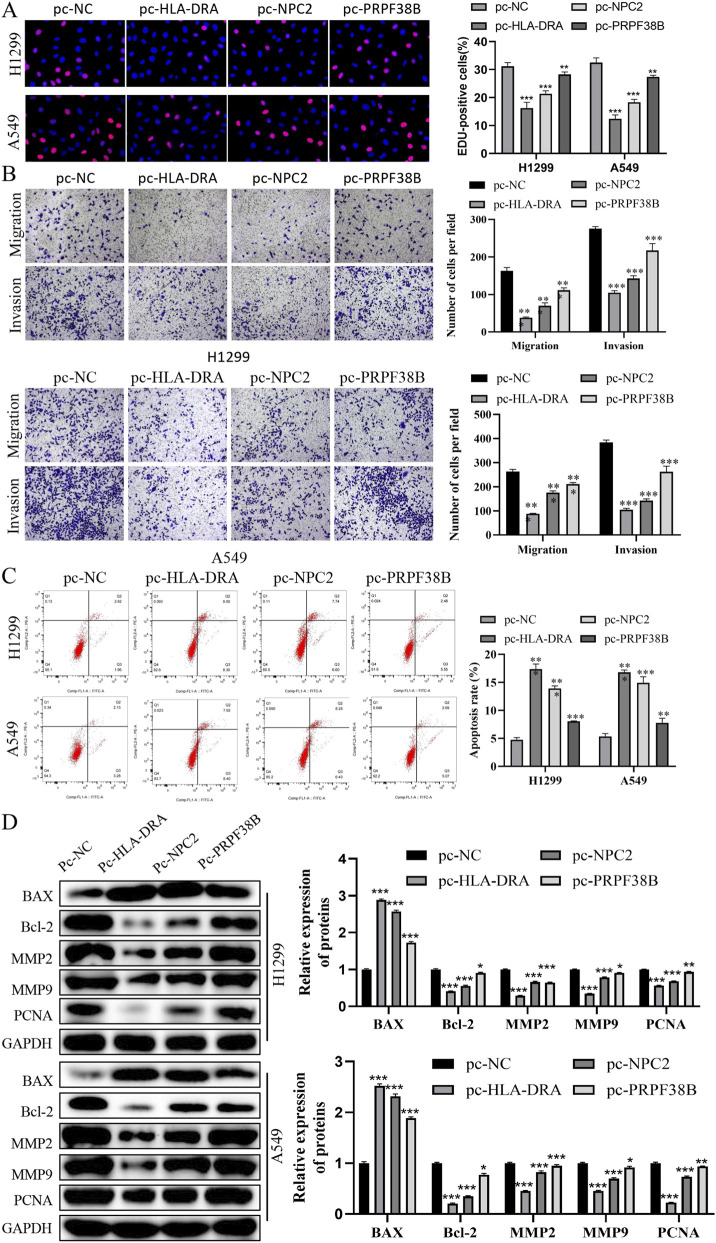
Effect of Overexpression of HLA-DRA, NPC2, and PRPF38 B on Proliferation, Migration, Invasion, and Apoptosis of Non-Small Cell Lung Cancer Cells. **(A)** EdU staining showing the effect of overexpression of HLA-DRA, NPC2, and PRPF38 B on the proliferation of H1299 and A549 cells. Quantitative analysis of the percentage of EdU-positive cells is shown on the right. **(B)** Transwell migration and invasion assays showing the effect of overexpression of HLA-DRA, NPC2, and PRPF38 B on the migration and invasion abilities of H1299 and A549 cells. Quantitative analysis is shown on the right. **(C)** Flow cytometry analysis showing the effect of overexpression of HLA-DRA, NPC2, and PRPF38 B on apoptosis in H1299 and A549 cells. Quantitative analysis of apoptotic cells (Annexin V-positive) is shown on the right. **(D)** Western blot analysis detecting the protein expression levels of BAX, Bcl-2, MMP2, MMP9, and PCNA in H1299 and A549 cells transfected with pc-HLA-DRA, pc-NPC2, and pc-PRPF38 B. The bar chart on the right shows the relative expression levels. ***p < 0.001, **p < 0.01, *p < 0.05 vs. pc-NC. Data are presented as mean ± SEM, and all experiments were performed in triplicate.

The overexpression efficiency of the plasmids (pc-HLA_DRA, pc-NPC2, and pc-PRPF38 B) in H1299 and A549 cells was then confirmed using RT-qPCR and Western blotting (Figures 7A,B). Overexpression of HLA-DRA, NPC2, and PRPF38 B significantly affected cell proliferation, migration, invasion, and apoptosis. The Edu assay results showed that overexpression of HLA-DRA, NPC2, and PRPF38 B significantly decreased the proliferation ability of H1299 and A549 cells compared to the control group (pc-NC) (Figure 8A). The findings of the Transwell experiment showed that these genes’ overexpression markedly reduced both cell types’ capacity for invasion and migration (Figure 8B). Overexpression significantly accelerated apoptosis in H1299 and A549 cells, according the flow cytometry data (Figure 8C). HLA-DRA, NPC2, and PRPF38 B overexpression was shown to decrease anti-apoptotic protein Bcl-2, increase pro-apoptotic protein Bax, and downregulate migration (MMP2, MMP9) and proliferation (KI-67, PCNA) proteins, according to Western blot analysis (Figure 8D). Our findings indicate that overexpression of PRPF38B, NPC2, and HLA-DRA suppresses cell invasion, migration, and proliferation while promoting apoptosis in H1299 and A549 cells. In addition, knockdown of PRPF38B, NPC2, and HLA-DRA increased the proliferation and migration abilities, and decreased the apoptosis rates of A549 cells (Supplementary Figure S6).

### 3.11 Effect of HLA-DRA, NPC2, and PRPF38 B overexpression on inflammation levels in H1299 and HCC827 cells and their impact on THP-1 macrophage polarization

**FIGURE 9:**
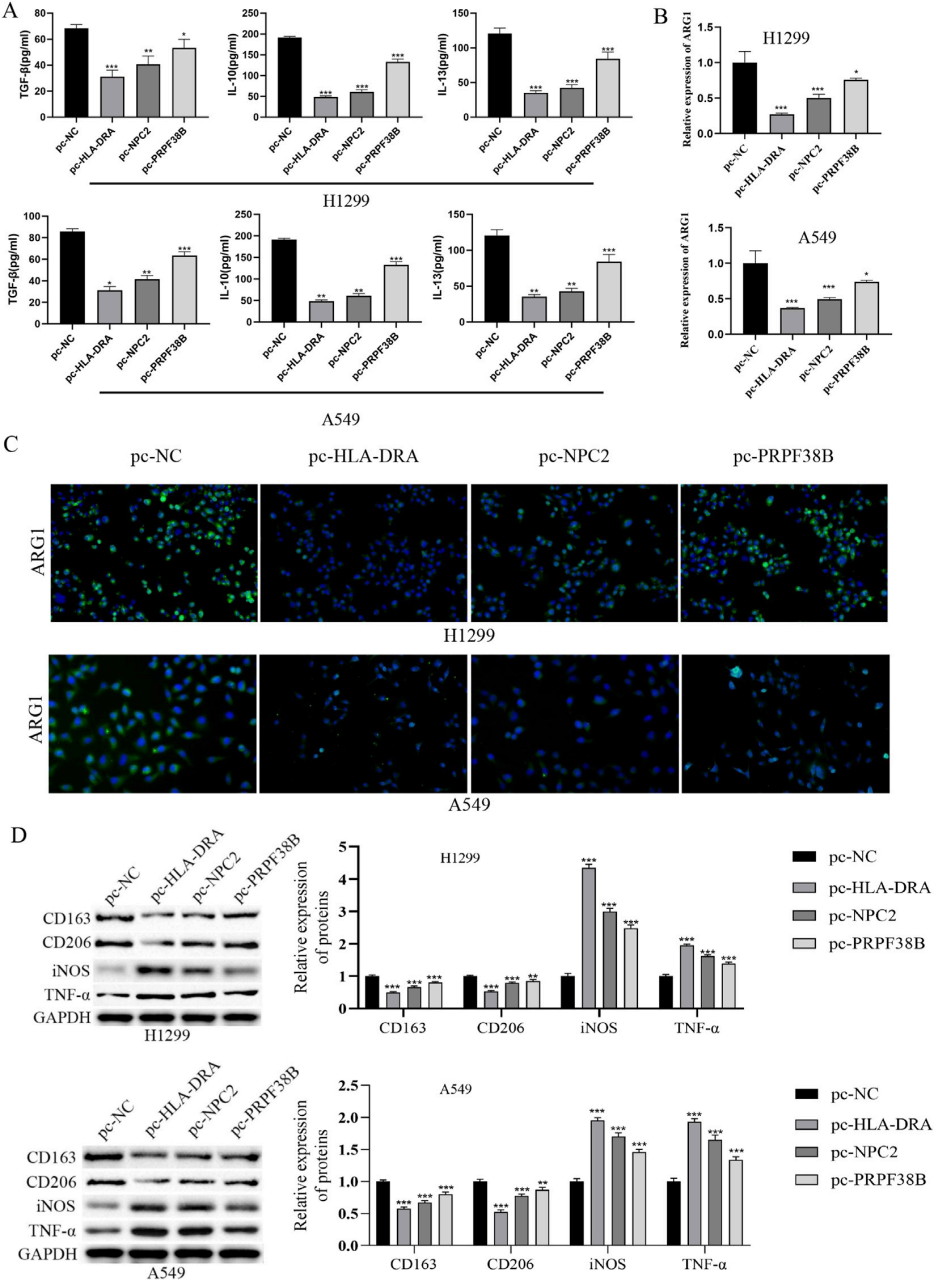
The Effect of Overexpression of HLA-DRA, NPC2, and PRPF38B on Inflammatory Levels in H1299 and HCC827 Cells and Polarization of THP-1 Macrophages. **(A)** ELISA detects the effect of overexpression of HLA-DRA, NPC2, and PRPF38 B on the secretion levels of TGF-β, IL-10, and IL-13 in H1299 and A549 cells. **(B)** PCR analysis shows the relative expression levels of ARG1 after overexpression of HLA-DRA, NPC2, and PRPF38 B. **(C)** Immunofluorescence staining detects the expression of ARG1 after overexpression of HLA-DRA, NPC2, and PRPF38 B. **(D)** Western blot was performed to detected the protein expressions of CD163, CD206, iNOS and TNF-α after overexpression of HLA-DRA, NPC2, and PRPF38 B. ***p < 0.001, **p < 0.01, *p < 0.05 vs. Pc-NC. The data are presented as mean ± SEM, and all experiments were performed in triplicate.

To further explore the immune regulatory roles of HLA-DRA, NPC2, and PRPF38 B in NSCLC, we overexpressed these genes in H1299 and A549 cells. ELISA results showed that overexpression of HLA-DRA, NPC2, and PRPF38 B significantly reduced the secretion levels of anti-inflammatory cytokines TGF-β, IL-10, and IL-13. Among them, the inhibitory effect of HLA-DRA was the most significant (Figure 9A).

Next, we co-cultured H1299 or A549 cells overexpressing HLA-DRA, NPC2, and PRPF38 B with THP-1 macrophages and analyzed the expression of the M2 polarization marker ARG1 in THP-1 macrophages by RT-qPCR. The results showed that overexpression of these genes significantly inhibited the expression of ARG1 in THP-1 macrophages, with HLA-DRA exhibiting the strongest inhibitory effect (Figure 9B). Immunofluorescence results further validated this finding, showing that in the overexpression groups of HLA-DRA, NPC2, and PRPF38B, the expression of ARG1 was significantly decreased, particularly in the HLA-DRA overexpression group, where there was almost no noticeable expression (Figure 9C).

In addition, we also analyzed the other macrophage polarization markers expressions. We found that HLA-DRA, NPC2, and PRPF38 B overexpression decreased the CD163 and CD206 protein expressions and increased the iNOS and TNF-α protein expressions (Figure 9D).

These results suggest that HLA-DRA, NPC2, and PRPF38 B effectively suppress the M2 polarization of THP-1 macrophages by reducing the secretion of inflammatory cytokines and inhibiting the expression of the M2 polarization marker ARG1. Notably, the inhibitory effect of HLA-DRA was the most pronounced.

## 4 Discussion

This study utilized LASSO regression to select 9 feature genes, including HLA-DRA, NPC2, and PRPF38B, and constructed an effective risk score model. These genes not only play important biological roles in NSCLC but are also closely associated with several key signaling pathways. For example, HLA-DRA is a gene primarily involved in antigen presentation and is highly expressed in immune cells, with its downregulation potentially leading to a weakened anti-tumor immune response (Rooney et al., 2015). NPC2’s upregulation is associated with metabolic pathways, suggesting that it may influence tumor growth by regulating metabolic reprogramming (Liao et al., 2015). By controlling RNA splicing and gene expression, PRPF38 B may contribute to the growth and migration of malignant cells (Sciarrillo et al., 2020). Further validation in this work indicated that overexpression of these genes significantly decreased NSCLC cell proliferation, migration, and invasion, and enhanced cell death, showing that these genes have protective roles in the incidence and progression of NSCLC.

The risk score model also revealed several key signaling pathways associated with tumor progression, including the P53 signaling pathway, PI3K-AKT-mTOR signaling pathway, and cell cycle regulation pathways. Apoptosis, DNA repair, tumor cell survival, and proliferation are all impacted by these pathways. For example, activation of the P53 signaling pathway can slow tumor progression by inducing apoptosis and inhibiting the cell cycle (Vousde and Prives, 2009; Levine and Oren, 2009; Aubrey et al., 2016). The PI3K-AKT-mTOR pathway regulates cell growth, metabolism, and survival, with its abnormal activation linked to cancer progression and resistance (Glaviano et al., 2023; Pavlova and Thompson, 2016). The cell cycle is a very well-organized series of processes that include cell division, growth, and DNA replication. The cell cycle regulatory pathway stops aberrant division, which is observed in the development of cancer, and regulates appropriate cell cycle progression (Malumbres and Barbacid, 2009; Hanahan and Weinberg, 2011). A theoretical basis for further investigation into the mechanistic roles of genes associated with risk scores is provided by these findings.

Notably, our analysis of immune checkpoints and immune cell infiltration revealed that high-risk patients exhibited significantly elevated expression of immune suppressor molecules including CTLA4, PDCD1, and TGFBR1, alongside increased levels of M0 macrophages and activated CD4 memory T cells, and reduced activated NK cells and CD8^+^ T cells. This pattern suggests that the high-risk group is in a state of immune exhaustion and immune tolerance rather than effective immune activation: the upregulation of immune checkpoints reflects the persistent stimulation of the immune system by tumor antigens, leading to impaired T cell function and an inability to mount an effective anti-tumor response; the accumulation of M0 macrophages—resting, unpolarized cells that can be skewed by tumor-derived signals toward an M2-like pro-tumor phenotype—further contributes to an immunosuppressive milieu. This finding provides a critical biological explanation for the poor prognosis of high-risk patients, linking the risk score model directly to the functional status of the NSCLC immune microenvironment.

The stability of the risk score model was confirmed in both the TCGA and GEO datasets, and ROC curves and Kaplan-Meier analysis were used to show how well the model predicted survival risk in patients with NSCLC. Specifically, the AUC values for 1-, 3-, and 5-year survival were all more than 0.65, indicating that the model had a high capacity for discriminating. Using the GDSC database, this study also examined the association between risk ratings and sensitivity to popular chemotherapeutic medications. It found that individuals in the high-risk category had considerably lower sensitivity to medications including cytarabine, axitinib, and AKT inhibitor VIII. The high-risk group was shown to be considerably enriched in pathways such MTORC1-SIGNALING, P53-PATHWAY, and DNA-REPLICATION using GSVA and GSEA analysis, and the changes in the activity of these pathways may contribute to the increased chemoresistance of tumor cells. Therefore, the risk score model not only provides a powerful tool for survival risk assessment in NSCLC but also offers valuable reference for optimizing chemotherapy regimens for patients.

This study identifies the possible functions of PRPF38B, NPC2, and HLA-DRA in NSCLC. These genes act as prospective therapeutic targets and prognostic indicators, offering new insights for precision therapy in NSCLC. They are also strongly linked to metabolic reprogramming and the tumor immunological microenvironment. There are several restrictions on this study, though. Firstly, although the functions of these genes were validated through cell experiments, *in vivo* model validation has not been completed. Secondly, the mechanisms of action of these genes, especially the specific impact of PRPF38 B’s RNA splicing function on tumor immunity and metabolic regulation, require further investigation. Future research could integrate single-cell sequencing technology and animal experiments to explore their specific mechanisms in the NSCLC microenvironment.

## Data Availability

The data that support the findings of this study are available from the corresponding author upon reasonable request.

## Supplementary material

Supplementary Figure S1 Quality Control and Data Integration in Single-Cell Analysis. **(A)** Violin plots of quality metrics (nFeature_RNA, nCount_RNA, percent.mt) for three samples (GSM4472055-2057). **(B)** Scatter plots showing correlations between percent.mt and nCount_RNA (left), and between nFeature_RNA and nCount_RNA (right), with Pearson coefficients indicated. **(C)** Top 10 highly variable genes ranked by standard deviation. **(D)** PCA colored by sample (orig.ident) showing variability and clustering. **(E)** Harmony integration corrects batch effects, with improved sample alignment in harmonized embeddings. **(F)** Elbow plot showing variance explained post-Harmony, used to select optimal principal components.

Supplementary Figure S2 Association of Risk Score with Clinical Indicators in NSCLC Prognosis. **(A)** Boxplot showing risk score distribution by survival status (alive vs. deceased). **(B)** Boxplot showing risk score distribution by T stage (T1–T4). **(C)** Boxplot showing risk score distribution by gender (male vs. female). **(D)** Boxplot showing risk score distribution by tumor stage (Stage I–IV). **(E)** Boxplot showing risk score distribution by N stage (N0–N3). **(F)** Boxplot showing risk score distribution by M stage (M0 vs. M1). **(G)** Boxplot showing risk score distribution by age (≤65 vs. >65). H-I: Univariate **(H)** and multivariate **(I)** Cox regression analyses showing HR and significance of risk score and clinical features (stage, gender, age, etc.).

Supplementary Figure S3 Immune Cell Composition and Immune Regulatory Gene Expression for NSCLC Risk Model. **(A)** Stacked bar chart showing the relative proportions of immune cells in the high-risk and low-risk groups. **(B,C)** Boxplots showing the expression levels of immune regulation-related genes in the high-risk and low-risk groups. ***p < 0.001, **p < 0.01, *p < 0.05 vs. LRisk.

Supplementary Figure S4 Expression, Pathway Enrichment, and Motif Analysis of Key Genes for NSCLC Risk Model. **(A)** Violin plot showing the expression distribution of key genes in the high-risk and low-risk groups. **(B)** GSEA enrichment analysis curve showing the pathways significantly enriched in the high-risk group, with the x-axis representing the ranked gene sets and the y-axis representing the enrichment scores.C: Motif annotation and enrichment analysis results for the model genes, including NES (Normalized Enrichment Score), AUC (Area Under the Curve), and associated transcription factors.

Supplementary Figure S5 Predictive Performance and Single-Cell Expression Analysis of Key Genes for NSCLC Risk Model. **(A)** ROC curve demonstrating the predictive performance of the nomogram model, including the AUC values for 1-year, 3-year, and 5-year survival predictions. **(B)** Decision Curve Analysis (DCA) showing the clinical net benefit of different models (risk score, clinical features, etc.) at various risk thresholds. **(C)** Violin plot showing the expression distribution of key genes across 8 cell types. **(D)** UMAP visualization of gene expression profiles. Each panel represents the expression of specific genes in clusters visualized using UMAP (Uniform Manifold Approximation and Projection). The color gradient from light blue to dark blue indicates the relative expression level of each gene, with darker shades representing higher expression. **(E)** Bubble plot displaying the expression level of key genes across different cell types. The bubble size indicates the proportion of model gene expression in specific cell types, while the color depth represents the average expression intensity.

Supplementary Figure S6 Effect of Knockdown of HLA-DRA, NPC2, and PRPF38B on Proliferation, Migration, and Apoptosis of Non-Small Cell Lung Cancer Cells. **(A)** EdU staining showing the effect of knockdown of HLA-DRA, NPC2, and PRPF38B on the proliferation of A549 cells. Quantitative analysis of the percentage of EdU-positive cells is shown on the right. **(B)** Transwell migration and invasion assays showing the effect of knockdown of HLA-DRA, NPC2, and PRPF38B on the migration abilities of A549 cells. Quantitative analysis is shown on the right. **(C)** Flow cytometry analysis showing the effect of knockdown of HLA-DRA, NPC2, and PRPF38B on apoptosis in A549 cells. Quantitative analysis of apoptotic cells (Annexin V-positive) is shown on the right. ***p < 0.001, **p < 0.01, *p < 0.05 vs. sh-NC. Data are presented as mean ± SEM, and all experiments were performed in triplicate.
